# Factors Associated With Weight, Length, and BMI Change in Adolescents' Offspring in Their First Year of Life

**DOI:** 10.3389/fped.2021.709933

**Published:** 2021-08-31

**Authors:** Reyna Sámano, Gabriela Chico-Barba, Hugo Martínez-Rojano, María Hernández-Trejo, Mery Birch, Maribel López-Vázquez, Gabriela Eurídice García-López, Jesús Díaz de León, Cynthia Vanessa Mendoza-González

**Affiliations:** ^1^Departamento de Nutrición y Bioprogramación, Instituto Nacional de Perinatología, Mexico City, Mexico; ^2^Programa de Doctorado en Ciencias Biológicas y de la Salud, Universidad Autónoma Metropolitana, Mexico City, Mexico; ^3^Escuela de Enfermería, Facultad de Ciencias de la Salud, Universidad Panamericana, Mexico City, Mexico; ^4^Sección de Estudios de Posgrado e Investigación, Escuela Superior de Medicina del Instituto Politécnico Nacional, Mexico City, Mexico; ^5^Coordinación de Medicina Laboral, Instituto de Diagnóstico y Referencia Epidemiológicos, Mexico City, Mexico; ^6^Neurobiología del Desarrollo, Instituto Nacional de Perinatología, Mexico City, Mexico; ^7^Licenciatura en Nutrición, Universidad del Valle de México, Mexico City, Mexico; ^8^Licenciatura en Nutrición, Universidad Tecmilenio, Mexico City, Mexico

**Keywords:** body weight, child growth, infant, pregnancy during adolescence, breastfeeding, Mexico

## Abstract

**Background:** Young maternal age is associated with negative outcomes at birth and with offspring's growth. In low- and middle-income countries, adolescents' offspring growth little has been studied.

**Objective:** To determine the association of maternal sociodemographic characteristics with weight, length, and BMI change in adolescents' offspring in their first year of life.

**Methods:** This is a one-year follow-up study that included adolescent mothers and their offspring from 2010 to 2017. The infant anthropometric variables were performed at birth, 3, 6, and 12 months. Maternal health, pregnancy, and social variables were evaluated as well as birth outcomes. Crude, percentage, Z score, and percentile changes of weight, length, and BMI were evaluated from birth to 1-year-old. Statistical analyses were adjusted by maternal chronological age, socioeconomic status, breastfeeding duration, the timing of introduction of complementary feeding, among other variables.

**Results:** We examined 186 dyads (mother-infant). The median maternal age was 15.5 years, and the mean pre-pregnancy BMI was 20. The mean gestational age was 39.1 weeks for infants, birth weight was 3,039 g, and length at birth was 49.5-cm. Maternal chronological age, the timing of introduction of complementary feeding, socioeconomic status, and maternal occupation were associated with offspring's weight gain at 12 months. Length gain was associated with exclusive breastfeeding. Socioeconomic status and occupation were associated with offspring's BMI change. When performing adjusted multivariable analyses, weight and length at birth were associated weight and BMI at 12 months.

**Conclusions:** Weight at birth may negatively predict infant's weight and BMI changes at 12 months, while length at birth may positively predict the changes. Maternal chronological age, socioeconomic level, occupation, and the timing of the introduction of complementary feeding were associated with the weight change. Only exclusive breastfeeding was associated with length Z-score change in adolescents' offspring in their first 12-months of life.

## Introduction

Adolescent pregnancy is associated with an increased risk of eclampsia, puerperal endometritis, systemic infections, maternal mortality, and preterm delivery compared to pregnant adults (≥20 years old) (1). Most of the research related to adolescent pregnancy and their offspring outcomes comes from high-income countries (2), even though teen pregnancy has higher rates in low- and middle-income countries (3). Pregnant adolescents may be at risk of being physically immature due to delayed completion of growth, especially when they have a pre-pregnancy body mass index (BMI) of underweight (4, 5).

The reasons for the adverse maternal and neonatal outcomes in adolescents are not clear yet, but it seems that inadequate/absent prenatal care is one of the main risk factors (6). Evidence shows that adverse perinatal outcomes in adolescents can be lowered with adequate prenatal care (7). In this sense, the lack of prenatal care in adolescent mothers has been related to a high risk of low birth weight and higher risk of perinatal mortality (8). Regarding neonatal outcomes, their anthropometric measurements can be influenced by several factors, like maternal age, pre-pregnancy weight, excessive gestational weight gain, and offspring's gender and gestational age (9–11).

Social and cultural factors are also associated with perinatal outcomes in pregnant adolescents. Many pregnant adolescents drop out of school, leading to a cycle of unemployment and poverty for life. These structural challenges resonate to promote the care of adolescent mothers. For example, the adolescents who go back to school often stop breastfeeding their children, contributing to the low rates of exclusive breastfeeding (8% at 6 months) (12). Therefore, it is predicted that adolescent mothers will have more difficulties in caring for their children, such as in breastfeeding practice and vaccines for children (2).

For the adolescents' offspring, the risk of perinatal morbidity and mortality, low birth weight, small for gestational age, and severe neonatal conditions is increased (13). Also, being an adolescent mother is associated with less knowledge about parenting and infant growth; for these reasons, adolescents may become less confident in their parental abilities, as well as being immature in their behavior and expressing more problematic parenting beliefs, and therefore, less able to meet the needs of their children (14, 15). On the other hand, as adolescents are still growing, there is a nutrient competition between the mother and the fetus (16).

Currently, very few longitudinal studies have investigated the after-birth growth of adolescents' offspring. Therefore, the purpose of the study was to determine the association of maternal sociodemographic characteristics with weight, length, and BMI change in adolescents' offspring in their first year of life.

## Materials and Methods

### Study Design and Subjects

We conducted a prospective cohort study at Mexico's Instituto Nacional de Perinatología (INPer) from 2010 to 2017. INPer is a public tertiary care institution that treats high-risk pregnancies of women who do not have formal employment or health insurance. The participants were 186 dyads of pregnant adolescents and their offspring. The children were born in the toco-surgical unit of INPer, and we followed them from birth to their first year of life. Sampling was non-probabilistic, and women who met the selection criteria were included consecutively. The participants were pregnant adolescents from 10 to 19 years old, primiparous, with a singleton pregnancy, with <20 weeks of gestation, and without chronic, metabolic, or genetic diseases. All participants signed informed assent, and their respective parents or guardians signed informed consent. We eliminated the participants that had gestational diabetes or hypertensive disorders during pregnancy. Also, dyads were eliminated if the offspring had a disease that affected growth, such as hypothyroidism, diabetes, heart diseases, neoplasms, or 21-trisomy. A structured questionnaire was used to gather sociodemographic data, like age, marital status, level of education, occupation, socioeconomic level, pre-pregnancy weight, history of diseases, and smoking and alcohol drinking habits.

### Procedures

#### Data Gathering

Trained health workers obtained the mothers' and their offspring's data using the questionnaire, clinical records, and performing interviews during the postnatal nutritional visits. We calculated the mother's chronological age at the moment of the delivery from the baseline data. We obtained the date of birth, sex, gestational age, birth weight, and length at birth directly from clinical records. We calculated gestational age at baseline evaluation or from the clinical record according to the last menstrual period. Neonatal outcomes were defined as follows: low birth weight <2,500 g, and small for gestational age as birth weight below the 10th percentile for specific gestational age and sex, according to the World Health Organization (WHO) criteria using AnthroPlus-WHO® (17).

#### Follow-Up Visits

Trained health professionals performed anthropometric measurements of the offspring at 3, 6, and 12 months. Birth weight was determined in grams on a pediatric digital scale Tanita 1583 (CMS Weighing Equipment Ltd., London, UK) with an accuracy of 0.01 kg. Length at birth was measured in centimeters with an infanto-meter Harpenden with an accuracy of 0.1 cm (CMS Weighing Equipment Ltd., London, UK). Length-for-age and weight-for-length were converted into sex-specific and gestational-age-adjusted Z-scores, respectively. Growth retardation was defined as a Z-score of length-for-age and weight-for-length and BMI below −2 SD, respectively (18).

Breastfeeding practice was prospectively assessed every 3 months during follow-up visits and was associate with growth variables: weight, length, and BMI. According to the definitions provided by the WHO (19), we evaluated any type of breastfeeding practice as the duration in months.

The socioeconomic level was considered a potential confounder; it was assessed with six dimensions of well-being within the household: Human Capital, Practical infrastructure, Connectivity and entertainment, Health infrastructure, Planning and future, and Basic infrastructure and space (20).

### Ethical Considerations

The Institutional Review Board and Ethics Committees from INPer approved the study with registration number 212250-49451. All data gathering was confidential. All participants received nutritional counseling to help them to improve their eating habits.

### Statistical Analysis

Frequencies and percentages were obtained for categorical variables. We calculated measures of central tendency and dispersion for numerical variables. We used the Kolmogorov-Smirnoff test to assess the distribution of numerical variables. Crude, percentage, Z score, and percentile changes of weight, length, and BMI were evaluated from birth to 1-year-old. Sociodemographic and clinical variables and breastfeeding duration were classified into two categories; then, weight, length, and BMI changes were compared between categories using a *t*-Student or a *U* Mann–Whitney test. Several univariate linear models were carried out to identify the variables that explained the weight, length, and BMI changes. These models were adjusted for possible confounding variables such as breastfeeding, maternal age, gestational age, educational lag, educational level, type of delivery, among others. All data were analyzed using the 21st version SPSS for Windows (IBM® Corp, Armonk, NY, USA). Statistical significance was declared at *p* < 0.05.

## Results

A total of 186 dyads (adolescent mothers and their children) participated in the study. All pregnancies were at term. Mean maternal age was 15.5 years and the median pre-pregnancy BMI was 20. Eighteen percent of the adolescents had a job or was a student; for educational level 27.4% had elementary school and 72.6% were at junior high, 36.6% of participants were ≤15 years, and 72.1% were from very low, low, and low-median socioeconomic levels.

The offspring' characteristics were as follows: 46% of the children were born by vaginal delivery, and 62% were female. The mean gestational age was 39.1 weeks of gestation, mean birth weight 3,039 g, and mean length at birth was 49.5 cm (Table 1).

**Table 1:** General characteristics of adolescent mothers and their offspring, *n* = 186 dyads.

	**Mean ± SD**	**Range**
**Adolescent mother characteristics**		
Age (years)	15.5 ± 1	12–17
Age at menarche (years)	11.5 ± 1	9–14
Height (cm)	155.5 ± 4	148–165.8
Pre-pregnancy BMI (kg/m^2^)	20	19–22
Gestational weight gain (kg) ^a^	12.4 (9.6–16.7)	−16–37
Educational lag (years) ^a^	2 (0-2)	0–6
	**Frequency (%)**	
**Sociodemographic characteristics**		
Occupation ^b^		
•Housewife	153 (82)	
•Had a job/student	33 (18)	
Socioeconomic level ^b^		
•Very poor	8 (4.3)	
•Very low	23 (12.4)	
•Low	103 (55.4)	
•Middle-low	38 (20.4)	
•Middle	14 (7.5)	
Marital status ^b^		
•Married/cohabiting	92 (49)	
•Single	94 (51)	
Education (years)	9 (8–9)	5–12
Type of delivery ^b^		
•Vaginal	85 (46)	
•Cesarean section	101 (54)	
**Offspring's feeding characteristics**		
Timing of the introduction of complementary feeding (months) ^a^	5 (4–6)	3–8
Exclusive breastfeeding (months) ^a^	3 (1–7)	0–12
**Offspring's characteristics at birth**		
Gestational age (weeks)	39.1 ± 1	37–41.6
Sex ^b^		
•Boy	71 (38)	
•Girl	115 (62)	
Anthropometric measurements		
•Weight (g)	3,039 ± 382	2,175–3,820
•Z-score weight	−0.5 ± 0.8	−2.8–1.18
•Percentile weight	34 ± 24	0.2–88.1
•Length (cm)	49.5 ± 1.5	46–53
•Z-score length	0.04 ± 0.8	−2.76–1.91
•Percentile length	51.1 ± 26	0.3–97
•BMI	12.4 ± 1	9.2–15.9
•Z-score BMI	−0.88 ± 1.2	−4–1.88
•Percentile BMI	29.6 ± 27	0–97

*SD, Standard Deviation; Range, Minimum–Maximum value*.

a *Median (25 percentile-75 percentile)*.

b *Frequency (percentage)*.

Boys and girls had similar birth weights (3,019 ± 344 vs. 3,071 ± 436 g, respectively; p = 0.366). In contrast, the length was greater among the boys than the girls were (49.7 vs. 49.2 cm, respectively; *p* = 0.028). There were no statistical differences in birth weight between mothers ≤15 years than mothers of 16 to 19 years (3,010 ± 401 vs. 3,055 ± 371, *p* = 0.435); neither did the length at birth (49.4 vs. 49.5 cm, *p* = 0.603). Low birth weight prevalence was 6.5% (12 out of 186), the timing of the introduction of complementary feeding was ≤5 months in 125 (67.2%) infants, and only 35.5% were exclusively breastfed for at least 6 months.

After 12 months of follow-up, there was only one case of low weight for age (0.5%). In contrast, 17 children (9.1%) were already in the percentiles of overweight or obesity. The rest of the children had an appropriate weight for their age. The offspring's mean weight at 12 months was lower in mothers ≤15 years compared to ≥16 years (9,307 *vs*. 9,739 g, *p* = 0.003). Infants who initiate complementary feeding before 6 months of age weighted less at 12 months of age than infants who initiated complementary feeding after 6 months of age (9,423 vs. 9,906, *p* = 0.001).

**Figure 1:**
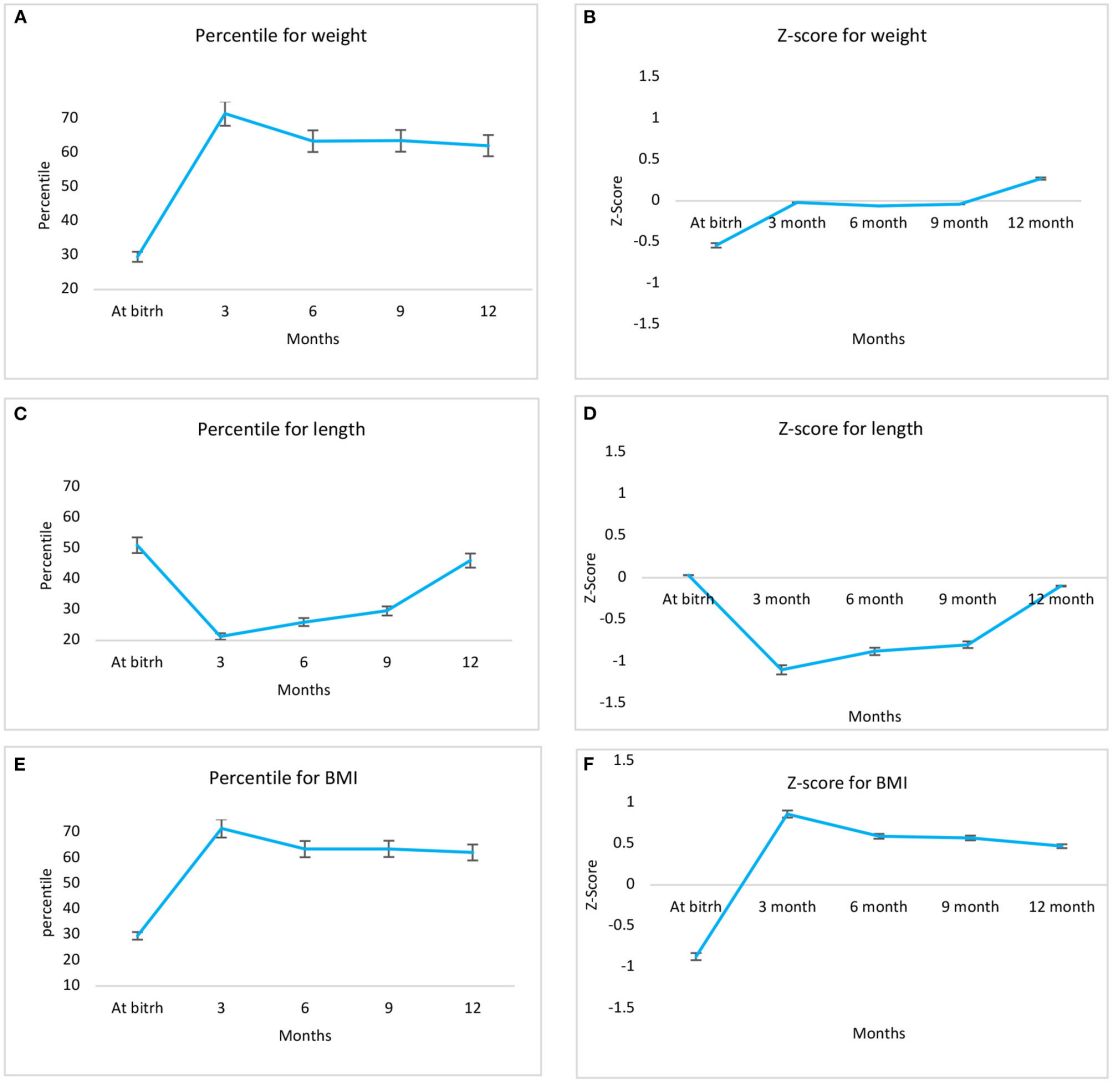
Trends in weight, length and BMI in percentiles **(left panel A,C,E)** and Z-score **(right panel B,D,F)**, from birth to 12 months in adolescents' offspring.

Figure 1 shows length and BMI trends (percentile and Z score) from birth to 12 months old.

Table 2 shows that adolescent's offspring from a low socioeconomic level had a mean weight change at 12 months of age lower than the children of mothers from a middle socioeconomic level. When comparing the offspring's weight at 12 months of age by mother's occupation, children from homemakers had higher weight than children of adolescent mothers who had a job or were students. Weight change during the first 12 months of life in adolescent mothers' offspring was different according to the mother's age, the timing of the introduction of complementary feeding, and the socioeconomic level.

**Table 2:** Weight change (delta) from birth to 12-months-old, according to maternal sociodemographic characteristics.

	**Grams**	**Percentage**	**Z-score**	**Percentile**
	**Mean ± SD**	**Mean ± SD**	**Mean ± SD**	**Mean ± SD**
**Weight**				
	6,544 ± 924	319 ± 42	0.82 ± 1	23.5 ± 31
**Maternal age**				
≤15 years (*n =* 68)	6,300 ± 879^*^	313 ± 45	0.69 ± 1	19 ± 33
≥16 years (*n =* 118)	6,684 ± 924	322 ± 41	0.89 ± 0.9	26 ± 29
**Timing of the introduction of complementary feeding**				
≤5 months (*n =* 125)	6,418 ± 912^*^	318 ± 45	0.77 ± 1	21.5 ± 32
≥6 months (*n =* 61)	6,802 ± 902	321 ± 38	0.90 ± 0.9	27.6 ± 28
**Breastfeeding at 6 months old**				
Exclusive (*n =* 66)	6,560 ± 902	317 ± 40	0.78 ± 1	24.5 ± 32
Non-exclusive (*n =* 120)	6,531 ± 942	320 ± 44	0.83 ± 1	22.8 ± 30
**Education level**				
Elementary school (*n =* 51)	6,559 ± 861	320 ± 40	0.88 ± 0.9	25.7 ± 25
High school (*n =* 135)	6,538 ± 948	319 ± 44	0.79 ± 1	22.7 ± 33
**Educational lag**				
No (*n =* 54)	6,364 ± 993	315 ± 45	0.74 ± 1	19 ± 35
Yes (*n =* 132)	6,617 ± 888	321 ± 42	0.85 ± 1	25 ± 29
**Socioeconomic level**				
Low (*n =* 134)	6,436 ± 906^*^	315 ± 42^*^	0.71 ± 1^*^	20.6 ± 30^*^
Middle (*n =* 52)	6,820 ± 922	330 ± 43	1.08 ± 1	31.0 ± 33
**Mother's occupation during pregnancy**				
Home wife (*n =* 153)	6,564 ± 949^*^	320 ± 42	0.85 ± 1^*^	24.5 (31)^**^
Student (*n =* 33)	6,456 ± 817	314 ± 43	0.68 ± 1	19.4 (33)
**Mother's occupation at 4 months postpartum**				
Home wife (*n =* 164)	6,592 ± 935^*^	320 ± 45	0.87 ± 1^*^	25.5 (31)
Student (*n =* 22)	6,185 ± 764	307 ± 42	0.43 ± 1	8.9 (32)
**Marital status**				
Single (*n =* 94)	6,423 ± 931	316 ± 44	0.72 ± 1	26.8 (33)
Married (*n =* 92)	6,667 ± 906	322 ± 40	0.91 ± 1	26.2 (28)

*SD, Standard Deviation*.

* *p < 0.050*

** *p ≤ 0.001*.

Regarding the length measurements, the change in length Z-score during the first 12 months of life in adolescent mothers' offspring differs only by the type of breastfeeding (0.050 ± 1 vs −0.27 ± 1, *p* = 0.040), the rest of sociodemographic variables did not have any statistical significance (Table 3).

**Table 3:** Change of length (delta) according to sociodemographic characteristics.

	**Centimeters**	**Percentage**	**Z-Score**	**Percentile**
	**Median (p25–p75)**	**Mean (SD)**	**Mean (SD)**	**Median p25–p75**
**Length (cm)**	24 (22.5–26)	149 (6)	−0.14 (1)	−5 (32)
	**Median (p25–p75)**	**Median (p25–p75)**	**Median (p25–p75)**	**Median p25–p75**
**Maternal age**				
≤15 years (*n =* 68)	23.5 (22–26)	148 (144-152)	−0.32 (−1.28-0.48)	−8.2 (−37–12)
≥16 years (*n =* 118)	24.5 (23-26)	149 (145-153)	−0.14 (−0.85-0.70)	−3.6 (−24–17)
	**Median (p25–p75)**	**Mean (SD)**	**Mean (SD)**	**Mean** **±** **SD**
**Timing of the introduction of complementary feeding**				
≤5 months (*n =* 125)	24.5 (22–26)	148 (6)	−0.21 (1.3)	−5 (31)
≥6 months (*n =* 61)	24.0 (22–26)	149 (6)	−0.02 (1)	−4 (36)
**Breastfeeding at 6 months**				
Exclusive (*n =* 66)	24.5 (23–26)	150 (6)	0.05 (1)^*^	0.4 (35)
Non-exclusive (*n =* 120)	24.0 (22–26)	148 (6)	−0.27 (1)	−8.4 (30)
**Education level**				
Elementary school (*n =* 48)	24.0 (22–26)	149 (6)	−0.08 (1)	−3.0 (34)
High school (*n =* 138)	24.0 (22–26)	149 (6)	−0.17 (1)	−5.7 (32)
**Educational lag**				
No (*n =* 54)	23.7 (22–26)	148 (7)	−0.30 (1)	−7.8 (35)
Yes (*n =* 132)	24.3 (22.5–26)	149 (6)	−0.01 (1)	−3.8 (32)
**Socioeconomic level**				
Low (*n =* 134)	24.0 (22.5–26)	149.1 (6)	−0.16 (1)	−4.9 (32)
Middle (*n =* 52)	24.5 (22.5–26)	149.4 (6)	−0.10 (1)	−5.2 (33)
**Mother's occupation during pregnancy**				
Home wife (*n =* 153)	24.5 (22–24.5)	149 (6)	−0.12 (1)	−4.2 (33)
Student (*n =* 33)	23.5 (23–25.7)	149 (5)	−0.24 (1)	−8.6 (26)
**Mother's occupation at 4 months postpartum**				
Home wife (*n =* 164)	24.0 (22.5–26.0)	149 (6)	−0.14 (1)	−4.6 (29)
Student (*n =* 22)	23.7 (22.7–26.2)	149 (6)	−0.20 (1)	−8.4 (29)
**Marital status**				
Single (*n =* 94)	24.0 (22–26)	148 (7)	−0.19 (1)	−5.4 (33)
Married (*n =* 92)	24.5 (22.5–26)	149 (6)	−0.10 (1)	−4.5 (32)

* *p < 0.040*.

Table 4 shows that the change in the BMI percentile during the first 12 months of life in adolescent mothers' offspring was statistically different by socioeconomic level and the mother's occupation during pregnancy.

**Table 4:** Change of BMI (delta) according to maternal sociodemographic characteristics.

	**BMI**	**Percentage**	**Z-Score**	**Percentile**
	**Median (p25–p75)**	**Median (p25–p75)**	**Median (p25–p75)**	**Median p25–p75**
**BMI**	4.6 (3.7-5.9)	137 (127-151)	1.1 (0.36-2.1)	31.5 (127-151)
**Maternal age**				
≤15 years (*n =* 68)	4.4 (3.6-5.8)	135 (127-152)	0.9 (0.3-2.3)	26 (5.5-56)
≥16 years (*n =* 118)	4.8 (3.7-5.9)	138 (128-151)	1.2 (0.4-1.2)	34 (10-56)
**Timing of the introduction of complementary feeding**				
≤5 months (*n =* 125)	1.6 (3.6-6.1)	137 (127-153)	1.0 (0.3-2.3)	29.8 (7.8-57.7)
≥6 months (*n =* 61)	4.8 (3.8-5.6)	138 (129-148)	1.3 (0.5-1.9)	34.8 (14-52.9)
**Breastfeeding at six months**				
Exclusive (*n =* 66)	4.1 (3.5–5.4)	134 (126–147)	0.8 (0.3–1.8)	24.8 (5.4–54.6)
Non-exclusive (*n =* 120)	4.9 (3.8–6.0)	139 (128–155)	1.3 (0.5–2.3)	34.4 (11–56.6)
**Education level**				
Elementary school (<8 y) (*n =* 48)	4.4 (3.8–5.6)	136 (128–147)	1.0 (0.3–1.8)	33.8 (128–147)
High school (>9 y) (*n =* 15)	4.8 (3.6–5.9)	138 (127–154)	1.1 (0.4–2.2)	31.4 (9.3–55.3)
**Educational lag**				
No (*n =* 54)	4.7 (3.6–6.0)	138 (127–152)	1.4 (0.4–2.3)	32.2 (11.3–57.1)
Yes (*n =* 132)	4.6 (3.7–5.8)	137 (127–150)	1.0 (0.3–2.0)	29.5 (8.4–55.1)
**Socioeconomic level**				
Low (*n =* 134)	4.4 (3.6–5.6)^*^	135 (127–148)	0.98 (0.3–1.8)	28.2 (6.2–52)^*^
Middle (*n =* 52)	5.2 (3.9–6.8)	141 (132–156)	1.5 (0.6–2.5)	39.6 (11.2–74)
**Mother's occupation during pregnancy**				
Homewife (*n =* 153)	4.7 (3.8–6.0)	137 (128–151)	1.1 (0.45–2.3)	36.6 (11.3–57.1)^*^
Student (*n =* 33)	4.2 (3.2–5.3)	133 (123–148)	0.7 (0.1–1.7)	18.6 (2.5–34.6)
**Mother's occupation at 4 months postpartum**				
Homewife (*n =* 164)	4.7 (3.7–5.9)	138 (128–151)	1.1 (0.4–2.2)	33.2 (10–56)
Student (*n =* 22)	4.1 (3.2–5.3)	132 (123–148)	0.7 (0.1–1.7)	19 (2.5–54)
**Marital status**				
Single (*n =* 94)	4.6 (3.7–5.8)	137 (128–150)	1.1 (0.3–1.9)	31.6 (5.6–55.1)
Married (*n =* 92)	4.7 (3.7–6.0)	137 (127–153)	1.0 (0.4–2.3)	31.4 (11.1–56.0)

* *p < 0.050*.

Only 35.5% of the children were exclusively breastfed for >6 months, and 67.2% began complementary feeding before 5 months of age. Weight, length, and BMI change at 12 months of the adolescent mothers' offspring were not statistically different according to the type of breastfeeding or the timing of the introduction of complementary feeding.

Tables 5, 6 show that weight and length at birth were the covariates that predicted the weight and BMI change at 12 months and maternal age, mother's occupation, and socioeconomic level, although with less power. Birth weight inversely predicted the anthropometric changes at 12 months according to all the performed models.

**Table 5:** Univariate general linear model to determine the predictor variables of weight change parameters.

**Covariates**	**Beta**	**Confidence interval 95%**	***p*-value**	**Eta square partial**	***R*^**2 adjusted**^**
**Weight change in z-score**					0.386
Weight at birth	−0.002	−0.002–0.001	0.000	0.362	
Length at birth	0.135	0.048, 0.221	0.002	0.050	
Mother's occupation	−0.99	−0.608, 0.011	0.059	0.020	
Maternal age	0.299	0.055, 0.543	0.017	0.031	
**Weight change in percentage (%)**					0.555
Weight at birth	−0.090	−0.103, −0.178	0.000	0.525	
Length at birth	4.739	1.672, 7.806	0.003	0.049	
Socioeconomic level	3.422	−3.313, 0.245	0.154	0.011	
Mother's occupation	−9.331	−20.17, 1.613	0.094	0.015	
Maternal age	10.686	1.950, 19.422	0.017	0.031	
**Weight change in percentile**					0.323
Weight at birth	−0.048	−0.059,−0.036	0.000	0.274	
Length at birth	0.539	−0774, 6.304	0.012	0.034	
Mother's occupation	−16.606	−26.51, −6.703	0.001	0.057	
Maternal age	8.948	1.139, 16.139	0.025	0.027	
**Weight change, crude (g)**					0.112
Weight at birth	−0.529	−0.913, −0.145	0.007	0.039	
Length at birth	150.992	57.63, 244.35	0.002	0.054	
Mother's occupation	−338.79	−671.65, −5.94	0.046	0.022	
Socioeconomic level	90.146	−55.33, 235.6	0.223	0.008	
Maternal age	347.571	81.62, 613.51	0.011	0.036	

**Table 6:** Univariate general linear model to determine the predictor variables of BMI change parameters.

**Covariates**	**Beta**	**Confidence interval 95%**	***p*-value**	**Eta square partial**	***R*^**2 adjusted**^**
**BMI change in z-score**					0.543
Weight at birth	−0.003	−0.004, −0.003	0.000	0.536	
Length at birth	0.471	0.357, 0.585	0.000	0.269	
Mother's occupation	−0.369	−0.776, 0.038	0.075	0.017	
Socioeconomic level	0.130	−0.046, 0.305	0.146	0.012	
**BMI change in percentage (%)**					0.561
Weight at birth	−0.046	−0.052, −0.040	0.000	0.557	
Length at birth	6.307	4.852, 7.762	0.000	0.288	
Socioeconomic level	1.852	−0.386, 4.090	0.104	0.015	
Mother's occupation	−3.656	−8.845, 1.533	0.166	0.011	
**BMI change in percentile**					0.369
Weight at birth	−0.067	−0.081, −0.054	0.000	0.351	
Length at birth	9.020	5.763, 12.278	0.000	0.142	
Mother's occupation	−13.98	−25.60, −2.36	0.019	0.030	
Socioeconomic level	3.522	−1.489, 8.489	0.167	0.011	
**BMI change, crude (kg/m** ^**2**^ **)**					0.408
Weight at birth	−0.004	−0.005, −0.003	0.000	0.398	
Length at birth	0.550	0.380, 0.721	0.000	0.184	
Mother's occupation	−0.520	−1.127, 0.087	0.093	0.016	
Socioeconomic level	0.211	−0.051, 0.473	0.113	0.014	

## Discussion

Birth weight is a health indicator of the offspring; it reflects the quality of nutrition during pregnancy, predicts immediate survival, and is essential to assess subsequent growth. In our study, the mean birth weight was 3,039 ± 382 g, which is very similar to that reported in children of adolescent mothers in Turkey, where the weight of the newborns was 2,934 g for mothers ≤15 years and 3,021 g for the children of mothers from 16 to 19 years (21). In contrast, the mean birth weight reported for Mexican children of adult mothers in Mexico City and the metropolitan area is 3,200 ± 373 g (22, 23). According to the previous data, the offspring in our study weighted 200 g less than these reports (24, 25). The offspring's anthropometric measurements can be influenced by several factors, including pre-pregnancy weight, excessive gestational weight gain, parity, gender, and gestational age (10).

Differences in pre-pregnancy body composition and gestational weight gain may have contributed to birth weight (10, 26). Studies show that adolescents tend to have low birth weight offspring, which suggests that the pregnant adolescent should receive prenatal care to look after the offspring's weight. Therefore, health care professionals should offer special attention to younger adolescents, who are the most likely to have a higher risk of adverse pregnancy outcomes. In addition, the children of adolescent mothers in developing countries are at a disadvantage at birth and during breastfeeding. In this sense, strategies should strengthen measures to prevent adolescent motherhood and to help to improve the nutrition and education of their children.

Also, offspring of mothers <15 years old have a higher risk of low birth weight. This finding is consistent with evidence showing that adolescent pregnancy is detrimental to the newborn (27–30). However, the low birth weight rate observed in our study is 6.5%, a rate that is lower than the established by the WHO (10% maximum) (31, 32). According to the WHO, in 2000, the prevalence of low birth weight in developed countries was close to 7%, while in South America, it was 9.6%. In 2015, this rate in Latin America and the Caribbean was 8.7% (33). The low rate of low birth weight that we obtained in our study could be attributed mainly to the quality and number of prenatal care visits (34, 35) received at INPer.

One of the main findings of this study was that birth weight and length at birth may predict the weight gain and BMI changes at 12 months old in the offspring of adolescent mothers. Nevertheless, in all statistical models performed, birth weight was the predictor variable on the anthropometric parameters' changes at 12 months old. Regarding the weight and length change during the first 12 months of life, both remained within the parameters of expected gain. However, the length change was slightly lower, although always within the parameters recommended by the WHO (17). Our results agree with that published by Chen et al. (36), who demonstrated that children from adolescent mothers had statistically significant lower weight (*p* < 0.001) and length (*p* < 0.001), although the speed and slope of growth were similar over time when compared with growth standards, and with data from the children of adult mothers. Wu et al. (37) also reported no significant statistical differences between children of adolescent mothers and children of adult mothers regarding children's growth and development parameters. These promising results are probably due to factors such as higher maternal education, as well as more favorable living conditions, as demonstrated by Luster et al. (38), in their study with children of teenage mothers and adult mothers from Michigan, USA.

Regarding exclusive breastfeeding, its frequency in our study group was 35.5%, which contrasts with that reported by Wambach and Cole (39), who discuss that adolescent mothers breastfeed for a shorter time than adult mothers. On the other hand, according to the National Health and Nutrition Survey 2012 (ENSANUT-2012) (40), in Mexico, the prevalence of exclusive breastfeeding in the general population was 18.5% in rural areas and 14.5% in urban areas. These figures are lower than what we observed in our study group. An explanation for these contrasting numbers is that INPer is a “child- and woman-friendly hospital,” where one of its primary purposes is to promote exclusive breastfeeding for at least 6 months.

Although the prevalence of exclusive breastfeeding was higher than that reported in urban areas of Mexico, it was not associated with weight gain in the adolescents' offspring at 12 months age, which is consistent with previous studies that show that offspring who are exclusively breastfed have a slower growth rate than that reported in infants who are fed with formula milk (41, 42).

Other research also has shown that the duration of breastfeeding is associated with slower growth during the first 12 months of the infant's life, which may contribute to the protective effects of breastfeeding against overweight and obesity at younger ages (43–46).

Our study highlighted that the timing of the introduction of complementary feeding was not associated with weight, length, and BMI changes of the children adolescent mothers at 12 months old. These findings are consistent with those reported by Dewey et al., in a systematic review of the efficacy and effectiveness of complementary feeding interventions in developing countries (47) as well as in the study by Woo et al., which included children of adolescent mothers from Mexico, the United States and China (48). In addition to the studies carried out by Briefel et al. (49), and by Chen et al. (36), in a cohort of adolescent mothers from Taiwan, in which the researchers discussed that this behavior might be due to the adverse socio-economic context in which adolescent mothers live, where the quality and quantity of food is compromised. However, one of the limitations of our study was the lack of information available on this topic. Also, the follow-up was only for 12 months to prove the impact of the timing of the introduction of complementary feeding and the practice of exclusive breastfeeding on the change in weight and length in the children of adolescent mothers.

Our finding that adolescent mothers who are engaged in household activities had offspring with a higher weight (9,648 ± 978) at 12 months old than offspring of adolescents who had a job or were students (9,648 ± 978 vs. 9,273 ± 903, respectively; *p* = 0.044). This finding adds up to the understanding that the care of the infant by its mother translates into better growth (36, 50). Therefore, others factors like socioeconomic level, mother's occupation, and the timing of the introduction of complementary feeding might be associated with weight, length, and BMI change in children of mothers of any age, not only in adolescents.

### Strengths and Limitations

The strength of the study is that the potential confounders were collected prospectively. Also, the longitudinal study design permits the assessment of causal relationships.

One of the study's limitations is that we do not have data regarding diet or nutritional supplementation use during pregnancy and other aspects of the offspring's diet (in addition to breastfeeding) and paternal factors, so it is not known if we can rule out residual confounders.

### Conclusions

Weight at birth negatively predicted offspring weight and BMI changes at 12 months, while length at birth positively predicted the changes. Only exclusive breastfeeding was associated with length Z-score change in adolescents' offspring in their first 12-months of life.

The offspring of adolescent mothers had lower weight and height, despite the fact that the speed and slope of the growth patterns were similar over time with the growth standards established by the WHO.

## Data Availability Statement

The raw data supporting the conclusions of this article will be made available by the authors, without undue reservation.

## Ethics Statement

The Institutional Review Board and Ethics Committees from INPer approved the study with registration number 212250-49451. The participant's legal guardian/next of kin provided written informed consent to participate in this study.

## Author Contributions

RS, HM-R, and GC-B: Conceptualization. RS, HM-R, GC-B, and MH-T: Data curation and Supervision. RS, HM-R, GC-B, MH-T, MB, and ML-V: Formal analysis. RS: Funding acquisition and project administration. RS, HM-R, GC-B, MH-T, MB, ML-V, and JD: Investigation. RS, HM-R, GC-B, MH-T, MB, ML-V, GG-L, JD, and CM-G: Methodology, writing – original draft and writing – review editing. RS, HM-R, and GC-B: resources and validation. RS, HM-R, GC-B, MH-T, and ML-V: Visualization. All authors contributed to the article and approved the submitted version.

## Conflict of Interest

The authors declare that the research was conducted in the absence of any commercial or financial relationships that could be construed as a potential conflict of interest.

## Publisher's Note

All claims expressed in this article are solely those of the authors and do not necessarily represent those of their affiliated organizations, or those of the publisher, the editors and the reviewers. Any product that may be evaluated in this article, or claim that may be made by its manufacturer, is not guaranteed or endorsed by the publisher.

## Abbreviations

BMI: Body Mass Index
ENSANUT: National Health and Nutrition Survey
INPer: Instituto Nacional de Perinatología
p-BMI: Pre-pregnancy Body Mass Index
SD: Standard Deviation
WHO: World Health Organization.
